# Revisiting volemic assessment via telemedicine: connecting traditional clinical needs with modern technology

**DOI:** 10.31744/einstein_journal/2026CE2185

**Published:** 2026-02-02

**Authors:** Eduardo Ferreira Amorim, Tarso Augusto Duenhas Accorsi, Carlos Henrique Sartorato Pedrotti

**Affiliations:** 1 Hospital Israelita Albert Einstein São Paulo SP Brazil Hospital Israelita Albert Einstein, São Paulo, SP, Brazil.

Dear Editor,

The widespread adoption of telemedicine has transformed healthcare delivery. However, the inability to perform hands-on physical examinations remains a significant limitation, particularly when accurate intravascular volume assessment is critical for conditions such as acute decompensated heart failure, sepsis, or renal failure.^(1)^ Conventional clinical signs—including elevated jugular venous pressure, peripheral edema, hepatojugular reflux, or pulmonary rales—are either difficult to reproduce consistently or challenging to assess reliably through standard videoconferencing. Moreover, even at the bedside, their individual diagnostic utility is limited.^(2)^

Evidence from emergency department studies of patients with dyspnea demonstrates that physical examination alone has limited effectiveness compared with a multimodal approach incorporating readily accessible ancillary tests. Integrating patient history, specific physical signs, chest X-rays, and plasma B-type natriuretic peptide (or NT-proBNP) levels substantially improves both sensitivity and specificity for diagnosing cardiogenic pulmonary edema. These findings underscore a broader principle relevant to telehealth: diagnostic accuracy in volume assessment requires supplementing remote visual inspection with objective, easily obtainable data.^(3)^

A significant advancement is remotely guided point-of-care ultrasound (tele-sonography). Handheld ultrasound probes, now widely available and increasingly affordable, can be operated by patients, family members, or local healthcare workers under real-time supervision by an off-site specialist. Respiratory variation in inferior vena cava dimensions and the collapsibility index provide robust estimates of right atrial pressure. Conversely, lung ultrasound measurement of B-lines offers a sensitive, semi-quantitative indicator of extravascular lung water. Temporal changes in B-line count have been validated as a reliable proxy for pulmonary congestion fluctuations in patients with acute and chronic heart failure, with independent prognostic significance. When images are streamed live or rapidly transmitted, the remote clinician effectively gains a "visual stethoscope" that substantially exceeds the diagnostic value of video-based inspection alone.^(4)^

Complementing tele-ultrasound, several continuous remote monitoring technologies provide objective longitudinal data on volume homeostasis. Non-invasive thoracic bioimpedance systems and remote dielectric sensing devices detect subtle lung water increases before symptom onset. More invasively, wirelessly implanted pulmonary artery pressure sensors—validated in the CHAMPION trial—enable preemptive diuretic and vasodilator titration, yielding significant reductions in heart failure hospitalizations. Meta-analyses of telemonitoring platforms confirm that algorithmic integration of daily weight, heart rate variability, bioimpedance, or intracardiac pressure trends facilitates earlier intervention and improved outpatient stability versus symptom-driven care. Through high-resolution video, digital stethoscopes, and shared-screen ultrasound probe control, an off-site intensivist or cardiologist can actively guide probe positioning, interpret findings in real time, and formulate management plans with accuracy approaching bedside consultation.^(5)^

In conclusion, although telemedicine cannot replicate the tactile components of traditional examination, a deliberate multimodal approach—combining guided point-of-care ultrasound, streamed physiologic data from wearable or implanted sensors, rapid biomarker analysis, and expert tele-supervision—effectively overcomes previous barriers to accurate remote volume assessment. This integrated model preserves and often surpasses the diagnostic and prognostic accuracy of traditional clinical signs alone, enabling safer, more proactive management of volume-sensitive conditions regardless of physical distance.

## Data Availability

The underlying content is contained within the manuscript.
